# Posttreatment surveillance intensity and overall survival in prostate cancer survivors (AFT-30)

**DOI:** 10.1093/jncics/pkae099

**Published:** 2024-10-09

**Authors:** Ronald C Chen, Ramsankar Basak, Stacie Dusetzina, Deborah S Usinger, Zahed Mohammed, Aaron D Falchook, Jessica R Schumacher, Amanda B Francescatti, Amanda Cuddy, George J Chang, Benjamin D Kozower, Caprice C Greenberg, Anne K Barber, Aaron J Katz

**Affiliations:** Department of Radiation Oncology, University of Kansas Medical Center, Kansas City, KS, USA; Department of Urology, University of North Carolina at Chapel Hill, Chapel Hill, NC, USA; Department of Health Policy and Vanderbilt Ingram Cancer Center, Vanderbilt University School of Medicine, Nashville, TN, USA; Lineberger Comprehensive Cancer Center, University of North Carolina at Chapel Hill, Chapel Hill, NC, USA; American College of Surgeons, Chicago, IL, USA; Memorial Healthcare System, Pembroke Pines, FL, USA; Department of Surgery, University of North Carolina at Chapel Hill, Chapel Hill, NC, USA; American College of Surgeons, Chicago, IL, USA; Department of Surgical Oncology, The University of Texas MD Anderson Cancer Center, Houston, TX, USA; Department of Surgical Oncology, The University of Texas MD Anderson Cancer Center, Houston, TX, USA; Department of Health Services Research, The University of Texas MD Anderson Cancer Center, Houston, TX, USA; Division of Cardiothoracic Surgery, Department of Surgery, Washington University School of Medicine, St. Louis, MO, USA; Department of Surgery, University of North Carolina at Chapel Hill, Chapel Hill, NC, USA; American College of Surgeons, Chicago, IL, USA; Department of Radiation Oncology, University of Kansas Medical Center, Kansas City, KS, USA; Department of Population Health, University of Kansas School of Medicine, Kansas City, KS, USA

## Abstract

**Background:**

Posttreatment surveillance affects millions of cancer survivors, but empiric data to guide clinical practice are lacking. This study assessed whether the intensity of surveillance testing after radical prostatectomy or radiation therapy for localized prostate cancer is associated with overall survival.

**Methods:**

Men diagnosed with localized prostate cancer between 2005 and 2010 who underwent radical prostatectomy or radiation therapy at a Commission on Cancer–accredited facility were randomly sampled. Primary data collected from 10 147 patients sampled across 1007 facilities were linked with existing data from the National Cancer Database. Analysis examined whether intensity of surveillance measured as the number of prostate-specific antigen (PSA) tests in the first year after primary treatment (categorized as 0-1 [low intensity], 2 [medium], or ≥3 [high intensity] PSA tests) was associated with overall survival. Secondary outcomes included recurrence-free survival (RFS) and subsequent use of imaging tests, biopsy procedures, and salvage treatment.

**Results:**

Median follow-up exceeded 8 years from prostate cancer diagnosis. Overall survival was not statistically significantly different across surveillance intensity groups among radiation therapy (*P* = .59) or radical prostatectomy (*P* = .29) patients. RFS was not statistically significantly different across surveillance intensity groups for radiation therapy (*P* = .13) patients but was for radical prostatectomy (*P* = .01) patients with high intensity associated with the worst RFS. In both treatments, higher surveillance intensity was associated with more procedures and salvage treatments.

**Conclusions:**

In patients with localized prostate cancer, more frequent PSA surveillance testing after radical prostatectomy or radiation therapy was associated with increased procedures and salvage treatments but not overall survival.

There are an estimated 3.5 million prostate cancer survivors in the United States in 2022 (1), and an estimated 299 010 are diagnosed in 2024 (2). After initial treatment, most commonly radical prostatectomy or radiation therapy, oncologic surveillance is required to monitor for prostate cancer recurrence. Published guidelines recommend routine prostate-specific antigen (PSA) testing as the primary surveillance modality, but the optimal intensity of posttreatment surveillance is unknown. More frequent surveillance may detect recurrence earlier and allow timely provision of salvage treatments, potentially improving survival. Conversely, greater surveillance frequency may lead to more frequent use of additional tests, procedures, and treatments, which may result in greater patient morbidity and increased costs. For patients with early stage cancers, it is possible these additional tests and procedures may increase risk of harm without providing a survival benefit.

Without high-quality empiric data on the optimal intensity of posttreatment surveillance in prostate cancer, guideline recommendations have varied considerably, ranging from every 3 months to every 12 months (3). In turn, these differences in the recommendations from professional guidelines may contribute to wide variation in clinical practice. The present study utilizes data collected from a large sample of men with prostate cancer from across the United States who received initial treatment with radiation therapy or radical prostatectomy. The primary goal of the study is to examine whether there is an association between frequency of posttreatment PSA surveillance testing and overall survival. We further examined if there is an association between frequency of PSA surveillance with additional procedures, imaging tests, and prostate cancer treatments.

## Methods

### Study design and data sources

Men diagnosed with localized (ie, nonmetastatic) prostate cancer in 2005-2010 and initially treated with radical prostatectomy or radiation therapy were identified from the National Cancer Database (NCDB), a joint project of the Commission on Cancer of the American College of Surgeons and the American Cancer Society. The NCDB integrates cancer registry records from Commission on Cancer–accredited hospitals and captures approximately 70% of all newly diagnosed cancers in the United States (4,5). Patient demographics (age at diagnosis, race, county of residence), cancer diagnosis [PSA level, Gleason score, and clinical stage at diagnosis; together used for National Comprehensive Cancer Network (NCCN) risk grouping (6)], comorbidities, and first course of treatment (type of treatment and dates) were obtained from predefined data elements in the NCDB that are recorded by certified cancer registrars at each facility via nationally standardized data item and coding definitions specified by the Facility Oncology Registry Data Standards (4,7).

The primary outcome of this study was overall survival. Treatment completion date and mortality date are predefined data elements collected by the NCDB.

### Additional primary data collection through the NCDB

Through the special studies mechanism, additional primary data collection can be performed for patients contained in the NCDB. For the current study, a random sample of up to 12 prostate cancer patients diagnosed in 2005-2010 was provided to each of the 1007 participating facilities for primary collection of data elements not routinely available in NCDB. Only patients diagnosed with localized prostate cancer who received radical prostatectomy or radiation therapy as primary treatment were eligible for sampling. For these randomly sampled patients, cancer registrars at the 1007 facilities abstracted additional data for posttreatment PSA testing, as well as recurrence, additional biopsy procedures, imaging, and prostate cancer salvage treatments received by each patient. These additional data were then merged into the NCDB data for this study’s analysis.

The study was determined exempt by the University of North Carolina at Chapel Hill institutional review board and did not require informed consent for the use of deidentified data.

### Statistical analysis

We defined surveillance frequency based on the PSA tests received in the first year after primary treatment and assessed the primary outcome (overall survival) and secondary outcomes (recurrence-free survival [RFS]; subsequent utilization of imaging tests, biopsy procedures, and prostate cancer treatment) after this exposure period to minimize the issue of immortality bias (8). Thus, patients who experienced an event during the first year after primary treatment were excluded from analysis for that specific outcome. Recurrence was defined by PSA level using standard definitions for radical prostatectomy and radiation therapy (9,10) or documented clinical recurrence, whichever occurred first.

The primary analysis was designed to compare overall survival among 3 surveillance intensity groups: patients who had 0-1 (low intensity) vs 2 (medium) vs at least 3 (high intensity) PSA tests. This corresponds to commonly recommended frequencies in clinical practice guidelines (every 12, 6, or 3 months, respectively). As a sensitivity analysis, surveillance intensity categories were also determined using PSA tests received in the first 2 years after primary treatment (low intensity 0-2, medium 3-5, high ≥6), with overall survival measured starting in the third year. Outcomes were observed through December 31, 2015.

Because the definition of recurrence differs for patients who received primary radical prostatectomy or radiation therapy (9,10), all analyses were run separately for prostatectomy and radiation patients. For patients after radical prostatectomy, biochemical recurrence is defined by a PSA level of 0.2 ng/mL (10). Biochemical recurrence after radiation therapy is defined by a rise of 2.0 ng/mL above the nadir level after treatment (9). Descriptive statistics were used to summarize receipt of biopsy procedures, imaging, and salvage treatments by patients in different surveillance frequency groups. For overall survival and RFS, we created Kaplan–Meier survival curves for each surveillance intensity group; differences in outcome across groups were evaluated using log-rank tests. *P* values were adjusted for multiple comparisons using the Šídák method (11). Further, the association between overall survival and posttreatment surveillance intensity was evaluated using multivariable Cox proportional hazards regression models. Covariates in the Cox regression models included age, race, NCCN risk group (low, intermediate, high), Charlson–Deyo comorbidity score (12), rurality of the patient’s residence (metropolitan, urban, rural) using 2013 rural-urban continuum codes (13), cancer center type (academic, comprehensive community, other) of the treatment facility where patients completed primary treatment, and census tract education level. As a sensitivity analysis, marginal Cox regression models were performed to account for the potential correlation of overall survival among patients treated within the same facility.

All tests of statistical significance were 2-sided and used an alpha level of 0.05. All analyses were performed using SAS software (version 9.4; SAS Institute Inc).

## Results

The overall study sample comprised 4865 patients who received radiation therapy (mean age = 66.4 years) and 5282 patients who received radical prostatectomy (mean age = 60.8 years) (Table 1). In both groups, patients were diverse in terms of race (27%-30% non-White) and represented different regions of the country. The vast majority (78%-80%) of patients were treated in nonacademic facilities. Demonstrating variations in practice, approximately one-third of radiation patients were included in each of the 3 surveillance intensity groups. For patients who received radical prostatectomy, the 3 intensity groups contained 31%, 25%, and 44% of patients. The characteristics of patients within each treatment group are further described by surveillance intensity in Supplementary Table 1 (available online).

**Table 1.** Characteristics of localized prostate cancer patients treated with primary radiation therapy or radical prostatectomy.

Characteristics	Radiation (n = 4865)	Radical prostatectomy (n = 5282)
Age, mean (SD), y	66.4 (6.3)	60.8 (6.9)
Age group, No. (%), y		
Younger than 55	261 (5)	1026 (19)
55-64	1371 (28)	2543 (48)
65-75	3233 (66)	1713 (32)
Race, No. (%)		
Black	714 (15)	615 (12)
Other^a^	710 (15)	786 (15)
White	3441 (71)	3881 (73)
NCCN risk group, No. (%)		
Low	1817 (37)	1717 (33)
Intermediate	1757 (36)	2240 (42)
High	1291 (27)	1315 (25)
Year of prostate cancer diagnosis, No. (%)		
2005	934 (19)	994 (19)
2006	1006 (21)	981 (19)
2007	1163 (24)	1166 (22)
2008	809 (17)	1021 (19)
2009	413 (8)	402 (8)
2010	540 (11)	718 (14)
Charlson–Deyo comorbidity score, No. (%)		
0	2571 (53)	3417 (65)
1	1206 (25)	1072 (20)
≥2	739 (15)	459 (9)
Region, No. (%)		
East	1104 (23)	1051 (20)
Midwest	1366 (28)	1557 (29)
South	1687 (35)	1753 (33)
West	708 (15)	921 (17)
County type,^b^ No. (%)		
Metropolitan	3740 (77)	4151 (79)
Urban	748 (15)	657 (12)
Rural	231 (5)	297 (6)
Facility type		
Academic	974 (20)	1178 (22)
Comprehensive Community Cancer Center	2476 (51)	2529 (48)
Other	1415 (29)	1575 (30)
Census tract % with less than high school education, No. (%)		
<14%	1674 (34)	2023 (38)
14% < 20%	1218 (25)	1243 (24)
20% < 29%	1088 (22)	1093 (21)
≥29%	741 (15)	767 (15)
Posttreatment surveillance intensity, No. (%)		
Low	1678 (34)	1614 (31)
Medium	1688 (35)	1336 (25)
High	1499 (31)	2332 (44)

a Other race includes American Indian and Alaska Native, Asian American, Hawaiian and Pacific Islander, other race, and unknown or missing race. NCCN = National Comprehensive Cancer Network.

b Based on 2013 Rural-Urban Continuum Codes (RUCC): metropolitan (RUCC 1-3), urban (RUCC 4-6), and rural (RUCC 7-9).

The median duration of follow-up from date of prostate cancer diagnosis was 8.1 years among radiation patients and 8.6 years among radical prostatectomy patients.

Table 2 summarizes the receipt of additional prostate cancer–related imaging, biopsy procedures, and salvage treatments in patients across surveillance intensity groups. Among patients who received primary radiation therapy, mean number of imaging tests per patient during surveillance was 2.3 in the low-intensity group, 2.3 in medium group, and 2.9 in high-intensity group (*P* = .002). Proportions of patients who received a biopsy procedure, any salvage treatment, or multiple types of salvage treatments also differed across groups, with higher surveillance intensity statistically significantly associated with more procedures and salvage treatments. Among patients who received primary radical prostatectomy, imaging, any salvage treatment, and multiple salvage treatments differed across groups. For example, 12.8% of patients in the low surveillance intensity group received any salvage treatment after radical prostatectomy, while 20.1% in the high-intensity group received salvage therapy.

**Table 2.** Receipt of additional prostate cancer–related imaging, biopsy procedures, and salvage treatments, by surveillance intensity^a^

	Posttreatment prostate-specific antigen surveillance intensity			*P*
Characteristic	Low	Medium	High	
Patients who received radiation therapy				
Imaging scans, mean number (SD)	2.3 (2.6)	2.3 (3.0)	2.9 (3.6)	.002
Biopsy, % of patients (95% CI)	4.6 (3.6 to 5.6)	4.6 (3.7 to 5.6)	7.1 (5.8 to 8.4)	<.001
Any salvage treatment, % of patients (95% CI)	7.3 (6.0 to 8.5)	8.2 (6.9 to 9.4)	10.3 (8.8 to 11.9)	.002
Multiple salvage treatments, % of patients (95% CI)	1.9 (1.3 to 2.6)	2.0 (1.4 to 2.6)	3.1 (2.3 to 4.0)	.024
Patients who received radical prostatectomy				
Imaging scans, mean number (SD)	2.1 (2.9)	2.2 (2.5)	2.6 (3.1)	.001
Biopsy, % of patients (95% CI)	3.0 (2.2 to 3.9)	3.1 (2.1 to 4.0)	3.1 (2.4 to 3.8)	.718
Any salvage treatment, % of patients (95% CI)	12.8 (11.2 to 14.5)	14.2 (12.3 to 16.1)	20.1 (18.5 to 21.7)	<.001
Multiple salvage treatments, % of patients (95% CI)	4.3 (3.3 to 5.3)	4.8 (3.6 to 5.9)	7.5 (6.4 to 8.5)	<.001

a CI = confidence interval.

**Figure 1.**
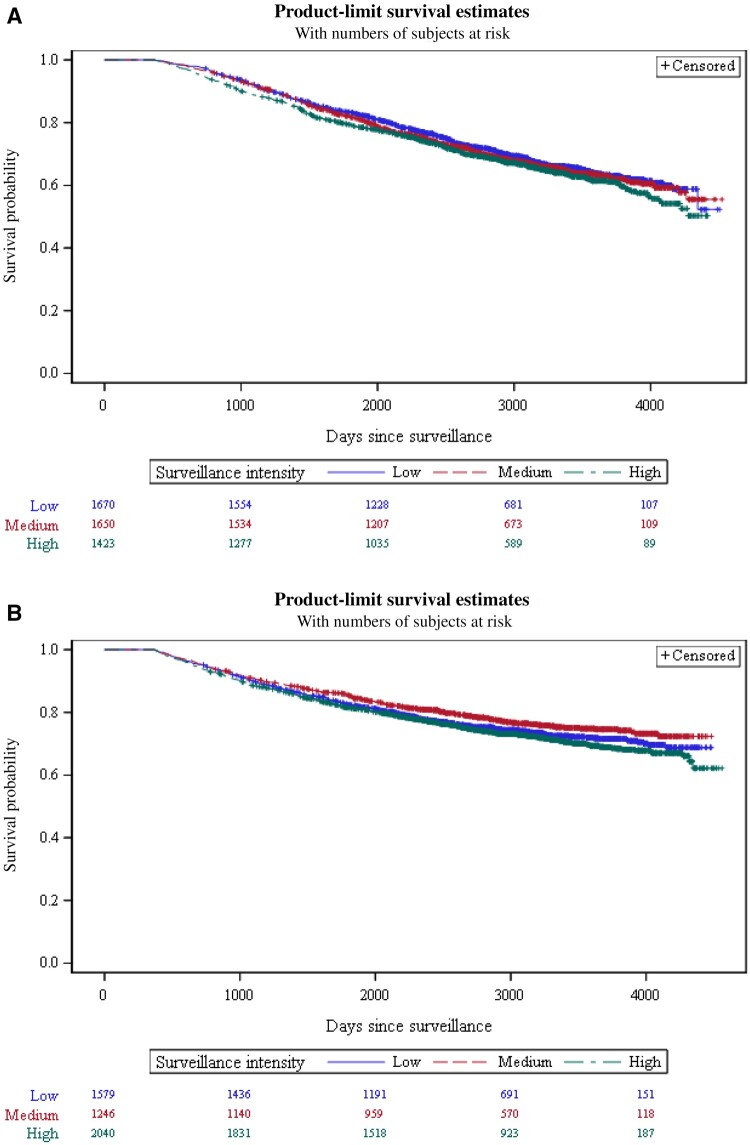
Recurrence-free survival by surveillance intensity for (**A**) radiation patients and (**B**) radical prostatectomy patients.

Figure 1 depicts the Kaplan–Meier RFS curves by surveillance intensity for patients treated with radiation therapy (Figure 1, A) or radical prostatectomy (Figure 1, B). Among radiation patients, 11% (95% confidence interval [CI] = 10% to 13%) of patients in the low-intensity group, 12% (95% CI = 10% to 13%) in the medium group, and 13% (95% CI = 11% to 15%) in the high-intensity group developed a recurrence or died. Among radical prostatectomy patients, 26% (95% CI = 24% to 28%, low intensity), 23% (95% CI = 21% to 25%, medium), and 28% (95% CI = 26% to 30%, high intensity) developed a recurrence or died. RFS was not statistically significantly different across surveillance intensity groups (log-rank test, χ^2^ = 4.02, *P* = .13) for radiation patients but was different for radical prostatectomy patients (log-rank test: χ^2^ = 8.47, *P* = .01).

**Figure 2.**
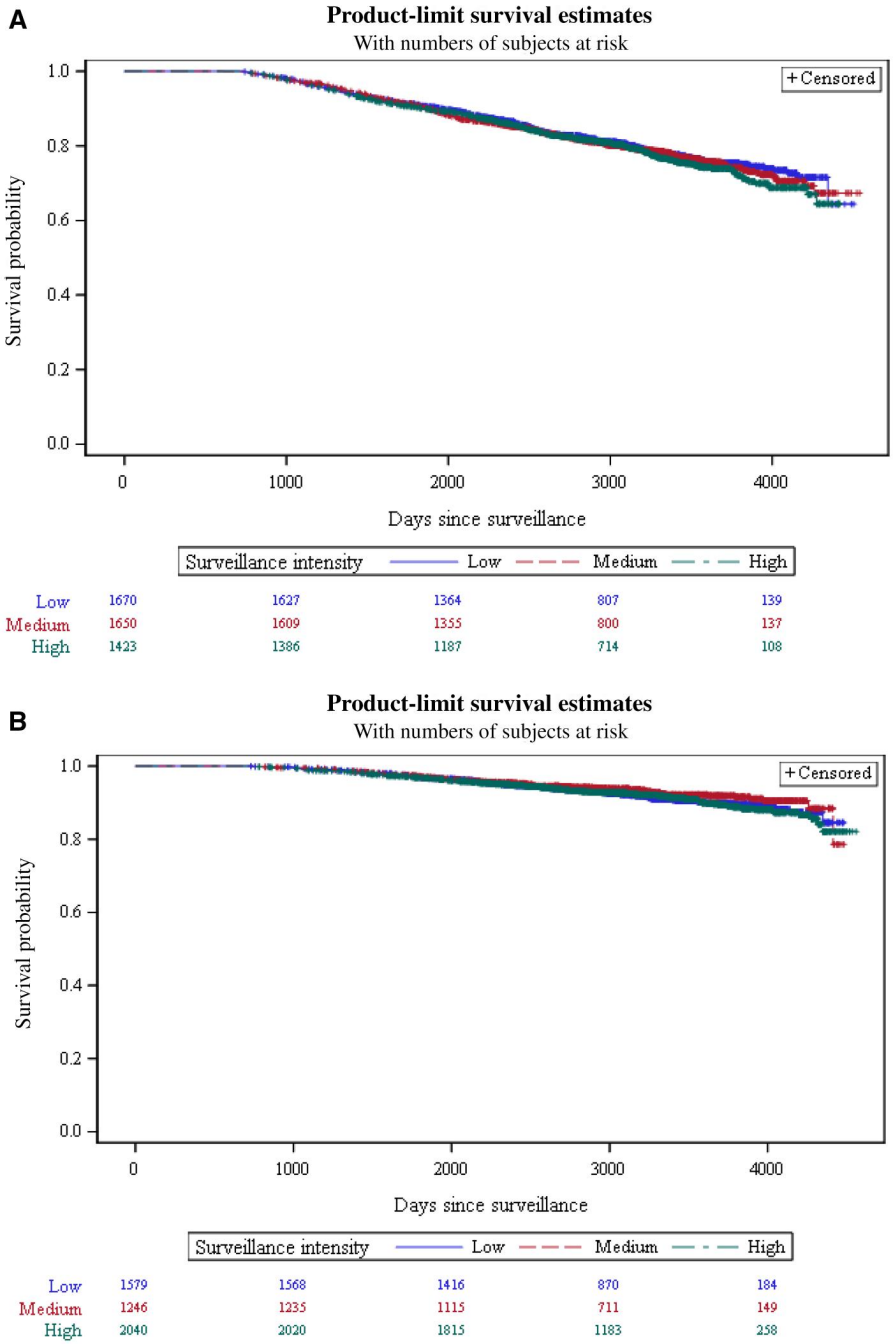
Overall survival by surveillance intensity for (**A**) radiation patients and (**B**) radical prostatectomy patients.

Kaplan–Meier overall survival curves by surveillance intensity are shown in Figure 2 for patients treated with radiation therapy (Figure 2, A) or surgery (Figure 2, B). Overall survival was not statistically significantly different across surveillance intensity groups among radiation (log-rank test: χ^2^ = 1.06, *P* = .59) or radical prostatectomy (log-rank test: χ^2^ = 2.46, *P* = .29) patients.

Multivariable Cox models showed no statistically significant association between surveillance intensity with overall survival in either radiation or radical prostatectomy patients (Table 3). Older age, higher NCCN risk group, and higher comorbidities were associated with increased mortality. Sensitivity analyses using marginal Cox regression models to account for clustering at the facility level produced similar results (Supplementary Table 2, available online). Another sensitivity analysis was performed, which grouped patients based on surveillance intensity in the first 2 years after treatment and evaluated overall survival starting in year 3 (Supplementary Table 3, available online), and again found similar results. Lastly, sensitivity analyses in which clinical T stage, Gleason score, and PSA level at diagnosis were included as individual covariates in Cox regression models (in place of NCCN risk group) produced similar findings (Supplementary Table 4, available online).

**Table 3.** Cox multivariable models for overall survival in radiation and radical prostatectomy patients

Characteristics	Radiation			Radical prostatectomy		
	No. of deaths	HR (95% CI)	*P*	No. of deaths	HR (95% CI)	*P*
Posttreatment surveillance intensity			.492			.309
Low	337	(Referent)		137	(Referent)	
Medium	358	1.06 (0.90 to 1.24)		103	0.96 (0.73 to 1.26)	
High	344	1.10 (0.94 to 1.30)		232	1.14 (0.91 to 1.42)	
Age, y		1.05 (1.03 to 1.06)	<.001		1.07 (1.05 to 1.08)	<.001
Race			.456			.351
Black	150	1.09 (0.89 to 1.33)		59	1.18 (0.86 to 1.63)	
Other^a^	151	0.93 (0.77 to 1.13)		62	0.89 (0.67 to 1.18)	
White	738	(Referent)		351	(Referent)	
NCCN risk group			<.001			<.001
Low	290	(Referent)		80	(Referent)	
Intermediate	382	1.29 (1.09 to 1.51)		186	1.71 (1.29 to 2.25)	
High	367	1.76 (1.49 to 2.07)		206	2.89 (2.18 to 3.82)	
Charlson–Deyo comorbidity score			<.001			<.001
0	379	(Referent)		233	(Referent)	
1	298	1.58 (1.35 to 1.85)		129	1.63 (1.30 to 2.03)	
≥2	311	2.91 (2.48 to 3.41)		90	2.55 (1.98 to 3.30)	
County type^b^			.072			.941
Rural	71	(Referent)		30	(Referent)	
Urban	174	0.83 (0.62 to 1.11)		60	0.94 (0.59 to 1.48)	
Metropolitan	756	0.75 (0.57 to 0.98)		365	0.99 (0.67 to 1.46)	
Facility type			.762			.671
Academic or other	516	(Referent)		231	(Referent)	
Comprehensive Community Cancer Center	523	0.98 (0.86 to 1.12)		241	1.04 (0.86 to 1.27)	
Census tract % with less than high school education			.204			.513
<14%	334	(Referent)		168	(Referent)	
14% < 20%	255	1.01 (0.86 to 1.20)		122	1.08 (0.84 to 1.37)	
20% < 29%	227	1.02 (0.85 to 1.22)		91	0.89 (0.67 to 1.18)	
≥29%	191	1.22 (1.00 to 1.50)		71	1.10 (0.81 to 1.49)	

a Other race includes American Indian and Alaska Native, Asian American, Hawaiian and Pacific Islander, other race, and unknown or missing race. CI = confidence interval; HR = hazard ratio; NCCN = National Comprehensive Cancer Network.

b Based on 2013 Rural-Urban Continuum Codes (RUCC): metropolitan (RUCC 1-3), urban (RUCC 4-6), and rural (RUCC 7-9).

## Discussion

Stakeholders and research prioritization efforts have identified posttreatment surveillance as a high-priority research topic, pointing to the lack of high-quality evidence needed to guide clinical practice (3,14,15). This is also an area that directly impacts millions of cancer survivors in the United States and worldwide. For prostate cancer, published guidelines recommend routine posttreatment surveillance using PSA testing; however, because of lack of empiric evidence, guideline recommendations vary from every 3 months to every 12 months surveillance, a 4-fold difference (3). This study was designed to directly address current knowledge gaps. Among 10 147 patients from across the United States with localized prostate cancer who received primary treatment with radiation therapy or radical prostatectomy, we found that higher intensity posttreatment PSA surveillance was associated with more imaging tests, biopsies (radiation patients only), receipt of any salvage therapy, and receipt of multiple salvage therapies. However, surveillance intensity was not associated with overall survival, a finding that was robust to sensitivity analyses.

Our findings appear to have face validity and are consistent with clinical expectations. Clinicians use PSA testing as the primary modality for posttreatment surveillance in prostate cancer survivors. Patients who receive more frequent PSA surveillance are naturally more likely to be detected with a PSA rise, leading to downstream additional testing with imaging and biopsies. Biopsies of the remaining prostate are commonly performed for radiation patients who are suspected to have a recurrence, as this is the most common site of recurrence; indeed, this study found that higher surveillance intensity was associated with more biopsies in radiation patients. Additionally, for patients who had surgical removal of the prostate, biopsies are expected to be less likely but could be performed for suspected metastatic areas. Thus, our observations of less frequent biopsies in radical prostatectomy patients (compared with radiation patients) and a lack of difference for biopsies across surveillance frequency groups among prostatectomy patients are not surprising.

We also found that higher-intensity surveillance was associated with RFS in radical prostatectomy but not radiation patients. This is likely explained by differences in the definitions for biochemical recurrence for prostatectomy and radiation patients. With a low PSA requirement to meet definition for recurrence, we found that 26%-28% of radical prostatectomy patients developed recurrence, and the highest intensity surveillance corresponded to the highest proportion of patients detected with recurrence. In addition, with a high PSA requirement to meet definition for recurrence, only 11%-13% of radiation patients were diagnosed with a recurrence; although directionally consistent with expectations, differences across surveillance intensity groups were not statistically significant.

We found one prior study examining the association between posttreatment surveillance intensity with overall survival (16). In a single-institution analysis of 832 men with localized prostate cancer after radical prostatectomy, radiation therapy, or primary androgen deprivation therapy, intensity of PSA surveillance was not associated with overall survival. Our results are overall consistent with this prior study. The efficacy and availability of multiple lines of salvage therapies are likely explanations for the lack of overall survival difference across patients who receive different frequencies of posttreatment surveillance.

In many ways, posttreatment surveillance for men who are not known to have a recurrence is analogous to prostate cancer screening for men who are not known to have prostate cancer. Prostate cancer screening is a subject of continued debate. Screening, usually done initially using PSA tests, leads to downstream biopsies, imaging tests, and treatments, which increase health-care costs and cause morbidity but may not increase overall survival in many men (17,18). The concepts of overdiagnosis and overtreatment of de novo prostate cancer are now widely recognized (18-20). Similarly, as our study shows, for men who have already received primary treatment for localized prostate cancer, highly intensive posttreatment PSA surveillance is associated with increased downstream tests and treatments but not improved survival. Thus, based on current guidelines and practice patterns, many men who have completed treatment for localized prostate cancer may be overdiagnosed and overtreated for recurrences that may not be life-threatening.

This study is limited by its nonrandomized design and therefore potential for confounding by indication. However, random assignment to different surveillance frequencies—especially wide-ranging frequencies from every 3 to every 12 months—may be difficult for patients to accept. In addition, sufficient enrollment and follow-up for such a trial would likely require more than 10 years overall to provide meaningful results. Thus, a cohort study such as ours likely provides the best achievable evidence to address the current knowledge gaps. We attempted to minimize the issue of confounding by indication by stratifying analyses by primary treatment type and adjusting for important measured covariates (eg, NCCN risk group) that may be associated with intensity of posttreatment surveillance. In addition, longer follow-up is necessary to fully assess mortality outcomes in localized prostate cancer. Other clinically meaningful outcomes such as quality of life, patient anxiety, and cost-effectiveness were not able to be assessed in this dataset. Our methods to account for potential immortality bias by excluding patients who experienced an event during the first year after primary treatment could have biased the outcome toward the null. We did not account for neoadjuvant or adjuvant androgen deprivation therapy, which can impact RFS and possibly overall survival outcomes.

An important strength of this study is the availability of a unique dataset that can examine these study questions. This dataset combined the strengths of the diversity and generalizability of patients included in the NCDB, where the vast majority of patients were treated in the community setting, with detailed medical record abstraction for additional data elements including surveillance intensity, downstream testing, recurrence, and salvage treatments. We are not aware of other cancer registry or large data sources with these data elements, which are required to assess the potential benefits and harms of posttreatment surveillance intensity. Another strength is the relatively long follow-up, which is necessary to examine overall survival in patients with localized prostate cancer.

In a large cohort of patients with localized prostate cancer who received radical prostatectomy or radiation therapy, more frequent posttreatment PSA surveillance was associated with increased imaging tests, biopsies, and salvage treatments but not overall survival. Future research may build on these findings by evaluating the impact of posttreatment surveillance intensity on patient-reported outcomes, such as anxiety, and financial toxicity, to further understand the extent to which more frequent posttreatment surveillance may affect additional meaningful patient outcomes.

## Supplementary Material

pkae099_Supplementary_Data

## Data Availability

Deidentified data will be made available upon request to the corresponding author.
