# Supplementary material for: Posttreatment surveillance intensity and overall survival in prostate cancer survivors (AFT-30)
Source: JNCI Cancer Spectr. 2024 Oct 9;8(6):pkae099. doi: 10.1093/jncics/pkae099 (PMC11560850; doi:10.1093/jncics/pkae099)
Supplement: pkae099_Supplementary_Data [file pkae099_supplementary_data.pdf]

Supplementary Tables

Supplementary Table 1. Characteristics of localized prostate cancer patients treated with primary radiation or radical prostatectomy, stratified by post-treatment surveillance intensity

Supplementary Table 2. Marginal proportional hazards models for overall survival in radiation and radical prostatectomy patients

Supplementary Table 3. Cox multivariable models for overall survival using 2 years of surveillance intensity

Supplementary Table 4. Cox multivariable models for overall survival using clinical T stage, Gleason score, and PSA level at diagnosis as individual covariates in place NCCN risk group

Supplementary Table 1. Characteristics of localized prostate cancer patients treated with primary radiation or radical prostatectomy, stratified by post-treatment surveillance intensity

	Radiation			Radical Prostatectomy		
**Characteristic**	**Low (n=1678)**	**Medium (n=1688)**	**High (n=1499)**	**Low (n=1614)**	**Medium (n=1336)**	**High (n=2332)**
**Age, mean (SD), y**	66.2 (6.3)	66.6 (6.3)	66.4 (6.4)	60.8 (6.8)	60.6 (6.8)	60.8 (7.0)
**Age group, n (%), y**						
<55	86 (5)	89 (5)	86 (6)	300 (19)	268 (20)	458 (20)
55-64	526 (31)	436 (26)	409 (27)	782 (48)	653 (49)	1108 (48)
65-75	1066 (64)	1163 (69)	1004 (67)	532 (33)	415 (31)	766 (33)
**Race, n (%)**						
Black	261 (16)	232 (14)	221 (15)	1142 (71)	984 (74)	1755 (75)
Other[a]	262 (16)	220 (13)	228 (15)	232 (14)	146 (11)	237 (10)
White	1155 (69)	1236 (73)	1050 (70)	240 (15)	206 (15)	340 (15)
**NCCN risk group, n (%)**						
Low	615 (37)	672 (40)	530 (35)	540 (33)	462 (35)	715 (31)
Intermediate	624 (37)	603 (36)	530 (35)	664 (41)	581 (43)	1005 (43)
High	439 (26)	413 (24)	439 (29)	410 (25)	293 (22)	612 (26)
**Year of prostate cancer diagnosis, n (%)**						
2005	351 (21)	308 (18)	275 (18)	345 (21)	243 (18)	406 (17)
2006	369 (22)	349 (21)	288 (19)	319 (20)	246 (18)	416 (18)
2007	376 (22)	432 (26)	355 (24)	351 (22)	289 (22)	526 (23)
2008	262 (16)	257 (15)	290 (19)	268 (17)	254 (19)	499 (21)
2009	148 (9)	134 (8)	131 (9)	133 (8)	95 (7)	174 (7)
2010	172 (10)	208 (12)	160 (11)	198 (12)	209 (16)	311 (13)
**Charlson-Deyo comorbidity score, n (%)**						
0	876 (58)	900 (57)	795 (56)	999 (68)	853 (68)	1565 (70)
1	387 (26)	431 (27)	388 (27)	341 (23)	271 (22)	460 (21)
2+	249 (16)	261 (16)	229 (16)	134 (9)	127 (10)	198 (9)
**Region, n (%)**						
East	443 (26)	362 (21)	299 (20)	294 (18)	285 (21)	472 (20)
Midwest	418 (25)	502 (30)	446 (30)	437 (27)	399 (30)	721 (31)
South	607 (36)	591 (35)	489 (33)	622 (39)	430 (32)	701 (30)
West	210 (13)	233 (14)	265 (18)	261 (16)	222 (17)	438 (19)
**County type,[a] n (%)**						
Metropolitan	1336 (82)	1283 (79)	1121 (77)	1300 (83)	1048 (81)	1803 (80)
Urban	221 (13)	275 (17)	252 (18)	203 (13)	175 (14)	279 (12)

Rural	69 (4)	74 (5)	88 (6)	69 (4)	69 (5)	159 (7)
**Facility type**						
Academic	290 (17)	361 (21)	323 (22)	287 (18)	313 (23)	578 (25)
Comprehensive Community Cancer Center	867 (52)	860 (51)	749 (50)	804 (50)	639 (48)	1086 (47)
Other	521 (31)	467 (28)	427 (28)	523 (32)	384 (29)	668 (29)
**Census tract % with less than high school education, n (%)**						
<14%	601 (37)	581 (35)	492 (34)	579 (37)	542 (42)	902 (40)
14% < 20%	401 (25)	463 (28)	354 (24)	356 (23)	303 (23)	584 (26)
20% < 29%	376 (23)	337 (21)	375 (26)	373 (24)	259 (20)	461 (20)
≥29%	252 (15)	261 (16)	228 (16)	255 (16)	194 (15)	318 (14)

[a] Other race includes American Indian/Alaska Native, Asian American, Hawaiian/Pacific Islander, other race, and unknown or missing race.
NCCN = National Comprehensive Cancer Network; SD = standard deviation.
[b] Based on 2013 Rural-Urban Continuum Codes: metropolitan (RUCC 1-3), urban (RUCC 4-6), and rural (RUCC 7-9).

Supplementary Table 2. Marginal proportional hazards models for overall survival in radiation and radical prostatectomy patients

	Radiation			Radical Prostatectomy		
**Characteristic**	**No. of deaths**	**HR (95% CI)**	***P* Value**	**No. of deaths**	**HR (95% CI)**	***P* Value**
**Post-treatment surveillance intensity**			0.500			0.322
Low	337	1 [Reference]		137	1 [Reference]	
Medium	358	1.06 (0.90, 1.24)		103	0.96 (0.74, 1.25)	
High	344	1.10 (0.94, 1.30)		232	1.14 (0.90, 1.44)	
**Age, y**		1.05 (1.03, 1.06)	<.001		1.07 (1.05, 1.08)	<.001
**Race**			0.550			0.336
White	738	1 [Reference]		351	1 [Reference]	
Black	150	1.09 (0.88, 1.34)		59	1.18 (0.86, 1.62)	
Other[a]	151	0.93 (0.74, 1.17)		62	0.89 (0.67, 1.17)	
**NCCN risk group**			<.001			<.001
Low	290	1 [Reference]		80	1 [Reference]	
Intermediate	382	1.29 (1.10, 1.51)		186	1.71 (1.29, 2.25)	
High	367	1.76 (1.49, 2.07)		206	2.89 (2.17, 3.84)	
**Charlson-Deyo comorbidity score**			<.001			<.001
0	379	1 [Reference]		233	1 [Reference]	
1	298	1.58 (1.36, 1.84)		129	1.63 (1.29, 2.05)	
2+	311	2.91 (2.49, 3.40)		90	2.55 (2.00, 3.27)	
**County type,[b]**			0.091			0.944
Rural	71	1 [Reference]		30	1 [Reference]	
Urban	174	0.83 (0.63, 1.09)		60	0.94 (0.59, 1.51)	
Metropolitan	756	0.75 (0.57, 0.97)		365	0.99 (0.67, 1.47)	
**Facility type**			0.805			0.697
Academic/Other	516	1 [Reference]		231	1 [Reference]	
Comprehensive Community Cancer Center	523	0.98 (0.84, 1.15)		241	1.04 (0.84, 1.29)	
**Census tract % with less than high school education**			0.277			0.503
<14%	334	1 [Reference]		168	1 [Reference]	
14% < 20%	255	1.01 (0.85, 1.21)		122	1.08 (0.84, 1.38)	
20% < 29%	227	1.02 (0.83, 1.24)		91	0.89 (0.67, 1.18)	
≥29%	191	1.22 (0.98, 1.53)		71	1.10 (0.80, 1.51)	

[a] Other race includes American Indian/Alaska Native, Asian American, Hawaiian/Pacific Islander, other race, and unknown or missing race. CI = confidence interval; HR = hazard ratio; NCCN = National Comprehensive Cancer Network; PSA = prostate specific antigen.
[b] Based on 2013 Rural-Urban Continuum Codes: metropolitan (RUCC 1-3), urban (RUCC 4-6), and rural (RUCC 7-9).

Supplementary Table 3. Cox multivariable models for overall survival using 2 years of surveillance intensity

	Radiation		Radical Prostatectomy	
**Characteristic**	**HR (95% CI)**	***P* Value**	**HR (95% CI)**	***P* Value**
**Surveillance intensity based on first 2 years post treatment**		0.159		0.445
Low (0-2)	1 [Reference]		1 [Reference]	
Medium (3-5)	1.12 (0.96, 1.31)		1.02 (0.81, 1.29)	
High (6+)	0.97 (0.79, 1.20)		1.16 (0.89, 1.51)	
**Age, y**	1.05 (1.03, 1.06)	<.001	1.07 (1.05, 1.08)	<.001
**Race**		0.429		0.349
White	1 [Reference]		1 [Reference]	
Black	1.07 (0.88, 1.30)		1.18 (0.86, 1.61)	
Other[a]	0.91 (0.75, 1.10)		0.89 (0.67, 1.18)	
**NCCN risk group**		<.001		<.001
Low	1 [Reference]		1 [Reference]	
Intermediate	1.29 (1.09, 1.51)		1.70 (1.29, 2.25)	
High	1.77 (1.50, 2.09)		2.86 (2.16, 3.80)	
**Charlson-Deyo comorbidity score**		<.001		<.001
0	1 [Reference]		1 [Reference]	
1	1.57 (1.35, 1.84)		1.62 (1.30, 2.02)	
2+	2.92 (2.50, 3.43)		2.55 (1.97, 3.29)	
**County type,[b]**		0.015		0.724
Rural	1 [Reference]		1 [Reference]	
Metropolitan or urban	0.66 (0.47, 0.92)		1.10 (0.62, 1.99)	
**Facility type**		0.598		0.689
Academic/Other	1 [Reference]		1 [Reference]	
Comprehensive Community Cancer Center	0.97 (0.85, 1.10)		1.04 (0.86, 1.26)	
**Census tract % with less than high school education**		0.117		0.510
<14%	1 [Reference]		1 [Reference]	
14% < 20%	1.04 (0.87, 1.23)		1.07 (0.85, 1.37)	
20% < 29%	1.05 (0.88, 1.25)		0.89 (0.67, 1.17)	
≥29%	1.26 (1.04, 1.54)		1.10 (0.82, 1.48)	

[a] Other race includes American Indian/Alaska Native, Asian American, Hawaiian/Pacific Islander, other race, and unknown or missing race. CI = confidence interval; HR = hazard ratio; NCCN = National Comprehensive Cancer Network; PSA = prostate specific antigen.

[b] Based on 2013 Rural-Urban Continuum Codes: metropolitan (RUCC 1-3), urban (RUCC 4-6), and rural (RUCC 7-9).

Supplementary Table 4. Cox multivariable models for overall survival using clinical T stage, Gleason score, and PSA level at diagnosis as individual covariates in place NCCN risk group

	Radiation			Radical Prostatectomy		
**Characteristic**	**No. of deaths**	**HR (95% CI)**	***P* Value**	**No. of deaths**	**HR (95% CI)**	***P* Value**
**Post-treatment surveillance intensity**			0.630			0.245
Low	337	[Reference]		137	[Reference]	
Medium	358	1.05 (0.90, 1.23)		103	0.97 (0.74, 1.27)	
High	344	1.08 (0.92, 1.27)		232	1.16 (0.93, 1.45)	
**Age, y**		1.05 (1.03, 1.06)	<.001		1.06 (1.05, 1.08)	<.001
**Race**			0.459			0.181
White	738	[Reference]		351	[Reference]	
Black	150	1.10 (0.90, 1.35)		59	1.24 (0.90, 1.71)	
Other[a]	151	0.94 (0.78, 1.14)		62	0.86 (0.64, 1.15)	
**Clinical T stage**			<.001			0.050
T1	616	[Reference]		262	[Reference]	
T2	368	1.27 (1.11, 1.46)		169	1.11 (0.90, 1.36)	
T3	55	1.45 (1.07, 1.96)		41	1.55 (1.09, 2.21)	
**Gleason**			<.001			<.001
≤6	410	[Reference]		153	[Reference]	
7	373	1.33 (1.14, 1.55)		179	1.66 (1.32, 2.10)	
≥8	256	1.64 (1.38, 1.96)		140	3.00 (2.32, 3.87)	
**PSA, ng/mL**	1039	1.00 (1.00, 1.01)	0.118	472	1.00 (0.99, 1.01)	0.679
**Charlson-Deyo comorbidity score**			<.001			<.001
0	379	[Reference]		233	[Reference]	
1	298	1.58 (1.35, 1.85)		129	1.62 (1.29, 2.02)	
2+	311	2.89 (2.47, 3.38)		90	2.56 (1.98, 3.30)	
**County type[b]**			0.033			0.967
Rural	71	[Reference]		30	[Reference]	
Urban	174	0.84 (0.63, 1.13)		60	0.94 (0.60, 1.50)	
Metropolitan	756	0.73 (0.56, 0.95)		365	0.95 (0.64, 1.41)	
**Facility type**			0.671			0.609
Academic/Other	516	[Reference]		231	[Reference]	
Comprehensive Community Cancer Center	523	0.97 (0.85, 1.11)		241	1.05 (0.87, 1.28)	

**Census tract % with less than high school education**			0.242			0.513
<14%	334	[Reference]		168	[Reference]	
14% < 20%	255	1.02 (0.86, 1.21)		122	1.09 (0.86, 1.39)	
20% < 29%	227	1.03 (0.86, 1.23)		91	0.89 (0.67, 1.19)	
≥29%	191	1.22 (0.99, 1.49)		71	1.12 (0.82, 1.52)	

[a] Other race includes American Indian/Alaska Native, Asian American, Hawaiian/Pacific Islander, other race, and unknown or missing race. CI = confidence interval; HR = hazard ratio; NCCN = National Comprehensive Cancer Network; PSA = prostate specific antigen.

[b] Based on 2013 Rural-Urban Continuum Codes: metropolitan (RUCC 1-3), urban (RUCC 4-6), and rural (RUCC 7-9).
